# A193 MICROBIOTA CHARACTERIZATION OF PATIENTS WITH INFLAMMATORY BOWEL DISEASE BASED ON SELF-REPORTED FOOD INTOLERANCES

**DOI:** 10.1093/jcag/gwae059.193

**Published:** 2025-02-10

**Authors:** R Dang, D Boron, J Linton, J Marshall, N Narula, A Caminero

**Affiliations:** McMaster University, Hamilton, ON, Canada; McMaster University, Hamilton, ON, Canada; University of Toronto, Toronto, ON, Canada; McMaster University, Hamilton, ON, Canada; McMaster University, Hamilton, ON, Canada; McMaster University, Hamilton, ON, Canada

## Abstract

**Background:**

Approximately, 20% of the world’s population experiences adverse reactions to different food items. Patients with inflammatory bowel disease (IBD) including Crohn’s disease (CD) and ulcerative colitis (UC) commonly exhibit more food intolerances. The dietary triggers and mechanistic pathways involved in food intolerances are unknown. As IBD patients present an altered intestinal microbiota, we hypothesized that IBD-related adverse reactions are associated with defective microbial metabolism.

**Aims:**

We aim to 1) understand dietary triggers and patterns of adverse reactions to foods in IBD and 2) characterize the intestinal microbiota of IBD patients based on food intolerance.

**Methods:**

127 participants (85 IBD and 42 healthy controls) were recruited from the Gastroenterology clinic and recruitment posters at McMaster University between August 2021 to September 2023. Inclusion criteria for IBD included a confirmed diagnosis of CD or UC and between 18 to 75 years old. All participants completed questionnaires related to food intolerances, symptoms, demographics, and provided a stool sample for 16S rRNA Illumina sequencing to determine microbial composition.

**Results:**

86% of IBD patients reported at least 1 food intolerance compared to 33% of controls. The mean number of food intolerances reported in patients with CD was 3.5 (SD=1.90), UC was 3.2 (SD=1.80), and controls was 1.3 (SD=0.83). Among IBD participants who reported a food intolerance, the common reactions were reported to dairy (72% CD and 73% UC), wheat (44% CD and 37% UC), and peanuts/tree nuts (28% CD and 30% UC). Regarding microbiota analysis, IBD patients had an altered fecal microbiota with lower alpha diversity (Shannon index) compared to healthy controls (p<0.0001). Alpha diversity was lower in IBD patients who reported 2 or more food intolerances compared to those who had none or 1. Also, specific adverse reactions such as dairy intolerance is associated with low alpha diversity in UC patients. IBD patients who reported a food intolerance had a higher abundance of Bacteroidota (p<0.05) compared to IBD with no food intolerance.

**Conclusions:**

Overall, IBD patients reported a high number of intolerances to foods including dairy, wheat, peanuts/tree nuts, and caffeine. IBD patients have a lower alpha diversity measure compared to controls, which was more pronounced in patients experiencing several food intolerances. Further research to understand these offending foods in intestinal inflammation could help guide dietary interventions for IBD patients.

**Funding Agencies:**

CCC

